# Changes in echocardiographic indices and left ventricular strain values by two-dimensional speckle-tracking echocardiography following pre-anesthetic oral pimobendan administration compared with intravenous pimobendan in dogs

**DOI:** 10.3389/fvets.2024.1394896

**Published:** 2024-06-03

**Authors:** Yijin Jeong, Bumseok Kim, Sung-Soo Kim, Kichang Lee, Hakyoung Yoon

**Affiliations:** ^1^Department of Veterinary Medical Imaging, College of Veterinary Medicine, Jeonbuk National University, Iksan, Republic of Korea; ^2^VIP Animal Medical Center, Seoul, Republic of Korea; ^3^Biosafety Research Institute and College of Veterinary Medicine, Jeonbuk National University, Iksan, Republic of Korea

**Keywords:** anesthesia, canine, echocardiogram, premedication, systolic function, speckle tracking

## Abstract

**Introduction:**

The effects of pre-anesthetic single-dose oral pimobendan during inhalational anesthesia, including the comparison with the effects of single intravenous pimobendan under anesthesia, remain unexplored. Therefore, this study aimed to determine changes in hemodynamic and echocardiographic parameters induced by pre-anesthetic administration of oral pimobendan under isoflurane general anesthesia and to compare them with those induced by intravenous pimobendan.

**Methods:**

Thirteen clinically normal dogs (4 laboratory and 9 client-owned dogs) with no clinical signs and not on any medical treatment were included. Anesthesia was performed three times: no pimobendan (Control), oral pimobendan (PIMO PO, 0.3 mg/kg), and intravenous pimobendan (PIMO IV, 0.15 mg/kg). Echocardiographic and hemodynamic parameters were monitored at 30-min intervals in all groups.

**Results:**

Compared to the Control group, end-systolic volume index (ESVI) and normalized left ventricular internal diameter at end-systole (LVIDSN) were significantly lower, and fractional shortening (FS) and ejection fraction (EF) were significantly higher in the PIMO PO and IV groups (*p* < 0.001). Global radial strain (GRS) was significantly higher in the PIMO PO and IV groups (*p* = 0.015).

**Conclusion:**

Under general anesthesia, oral pimobendan preserved LV systolic and myocardial function in a manner comparable to intravenous pimobendan. Pre-anesthetic administration of oral pimobendan can be used to compensate for cardiac systolic function in dogs who require therapeutic and diagnostic procedures under general anesthesia with potential risk of circulatory failure.

## 1 Introduction

General anesthesia is widely used for various diagnostic tests and therapeutic surgical procedures, despite frequent cardiorespiratory complications such as cardiac dysrhythmias, hypotension, hypoxemia, and hypoventilation (1, 2). Diminished systemic circulation due to decreased cardiovascular function can induce organ hypofunction, such as in the kidneys (3) and pancreas (4, 5). Fluid therapy, anticholinergics, vasoconstrictors, or positive inotropes mitigate this diminished systemic circulation and the related consequences (1). Pimobendan, an FDA-approved inotropic agent used in congestive heart failure (CHF), functions via a mechanism of action that increases calcium sensitivity and inhibits phosphodiesterase 3, with resultant cardiac contractile and vasodilatory effects (6). Pimobendan is an effective treatment for CHF secondary to chronic valvular heart disease (7–9) and dilated cardiomyopathy (DCM) (10). Recent studies have shown that single-dose intravenous pimobendan administration improves cardiac function by increasing systolic output and left ventricle (LV) contractility in dogs under inhalational anesthesia (11, 12). Another study compared the efficacy and hemodynamic effects of intravenous and intramuscular pimobendan in anesthetized dogs (13). Although the short- and long-term echocardiographic changes induced by oral pimobendan have been studied in unanesthetized dogs (14–16), no study has evaluated the effects of single-dose oral pimobendan premedication during general anesthesia in dogs. There are also no studies comparing oral pimobendan to intravenous pimobendan under general anesthesia.

Conventional echocardiography is the most commonly used non-invasive method to assess the systolic cardiac function of the heart. Two-dimensional speckle-tracking echocardiography (2D-STE), which analyzes myocardial function by measuring myocardial deformation, is used in human and veterinary medicine (17, 18). In veterinary medicine, normal values in clinically healthy dogs (19–21) and in dogs with cardiac diseases, such as myxomatous mitral valve disease (22), dilated cardiomyopathy (23), and patent ductus arteriosus, are known. Myocardial strain using 2D-STE is useful in evaluating the LV systolic function compared to invasive measurements using cardiac catheters (24).

This study aimed to determine the changes in echocardiographic parameters and strain values during inhalational anesthesia with pre-anesthetic oral pimobendan administration and to compare them with intravenous pimobendan administration. Furthermore, the study evaluated the usefulness of oral single-dose pimobendan administration in anesthetized dogs.

## 2 Materials and methods

### 2.1 Animals

This study comprised 13 dogs (9 males and 4 females), aged 7.62 ± 1.01 years and weighing 10.77 ± 1.24 kg of the following breeds: Beagle (*n* = 6), Mixed (*n* = 3), Poodle (*n* = 3), and Chow (*n* = 1). Four dogs were laboratory dogs (from January 2018 to February 2018), and the remaining 9 were client-owned dogs that presented for dental radiography at the Jeonbuk National University Animal Medical Center from January 2023 to April 2024. They were enrolled after obtaining the consent from their owners and underwent additional echocardiography under anesthesia. Their history-taking, physical examination, and blood analysis (complete blood count, serum biochemistry) performed prior to anesthesia did not show any abnormalities. Pre-anesthetic transthoracic echocardiography revealed subclinical mild mitral regurgitation (*n* = 4), physiologic pulmonic regurgitation (*n* = 2), and physiologic tricuspid regurgitation (*n* = 3). These regurgitations observed before anesthesia were observed during anesthesia. None of the subjects were undergoing treatment during the experiments. This study was approved by the Institutional Animal Care and Use Committee of Konkuk University (KUIACUC-KU18073, 2018) and Jeonbuk National University (JBNUNON2022-083, 2022).

### 2.2 Study design

**Figure 1:**
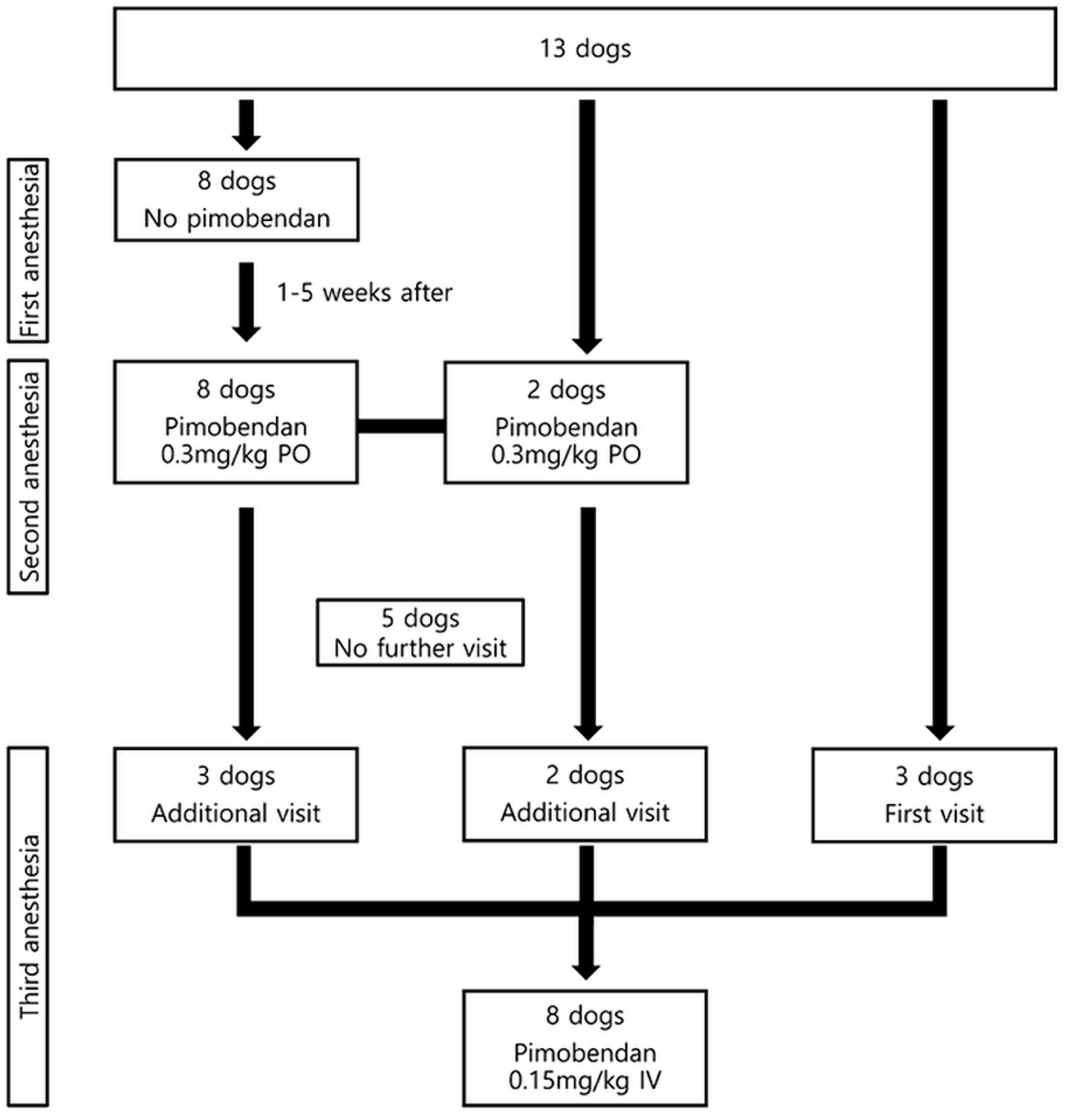
Flow diagrams depicting the anesthesia protocols and time intervals.

An overview of the anesthetic process and subject disposition in this double-blinded, randomized parallel group clinical trial is shown in Figure 1. First and second anesthesia were performed to determine the effect of oral pimobendan on echocardiographic parameters during anesthesia. The first anesthesia (Control group) was performed without pre-anesthetic pimobendan administration, and the second anesthesia (PIMO PO group) was performed with 0.3 mg/kg oral pimobendan (Vetmedin 5 mg tablet, Boehringer Ingelheim, Mexico City, Mexico) administration 30 min prior to propofol induction. The interval between the two anesthesia experiments was 1–5 weeks, which was considered an adequate interval for wash-out of the pharmacological effects of the first anesthesia (25, 26). Baseline values of heart rate (HR), systolic blood pressure (SBP), and echocardiography were evaluated before the pimobendan administration of anesthesia. Serial transthoracic echocardiography was performed at 30 and 60 min after induction (T30 and T60), and we compared the changes in the echocardiographic parameters between the Control and PIMO PO groups. The third anesthesia (PIMO IV group) was administered to compare the effects of oral and intravenous pimobendan administration in 8 dogs, which consisted of 5 from the previous anesthesia groups and 3 new dogs that had never undergone the study-related anesthesia. The measurement intervals and methods were modified and adapted from previous studies (13, 27). Accordingly manufacturer’s instructions, 0.15 mg/kg IV pimobendan (Vetmedin 5 mL solution for injection, Boehringer Ingelheim, Barcelona, Spain) was injected 10 min after propofol induction in the third anesthesia.

### 2.3 Anesthesia and preparation

Anesthesia in all dogs was induced by a slow intravenous injection of 6.0 mg/kg propofol (Freefol-MCT inj. 1%, Daewon Pharm, Seoul, South Korea). Endotracheal intubation was performed, and anesthesia was maintained with isoflurane (Ifran, Hana Pharm, Hwaseong, South Korea) to 100% O_2_. The concentration of isoflurane during anesthesia was individualized to 2–3%, and a 1–1.5 minimum alveolar concentration was maintained. The HR, saturation of percutaneous oxygen (SpO_2_), and expiratory terminal PaCO_2_ (EtCO_2_) were continuously monitored using tongue-mounted pulse oximetry, and remained above 98%, whereas EtCO_2_ was maintained at 35–45 mmHg. Indirect SBP was measured by a Doppler flow detector before anesthesia and at 30-min intervals after induction. Dehydration was not detected in any dog prior to anesthesia, and fluid therapy was not administered during any anesthesia. After the termination of anesthesia, adverse events, including vomiting, were monitored for 24 h in the hospital, and fluid therapy was continued until discharge. Three days after discharge, administrators and owners were contacted to ascertain any abnormal findings.

### 2.4 Echocardiography

Echocardiographic data (images and clips) were acquired by a same radiologist with 10 years of experience. All parameters were subsequently measured from the collected images and clips, and calculated by a single radiology resident using the same ultrasound machine and confirmed by the radiologist. Each view was recorded for more than three cardiac cycles, and the same view was recorded at least three times. Transthoracic echocardiography was performed using the Aplio i800 (Canon Medical Systems Corporation, Otawara, Japan) with a 6S3 sector transducer (3.0–8.2 MHz) and the EPIQ 7G (Philips Healthcare, Bothell, Washington, USA) with a S8-3 sector transducer (3–8 MHz). Moreover, continuous lead II ECG monitoring was performed. The right parasternal short-axis 2D view was used to measure the left atrial-to-aortic root (LA/Ao) ratio. M-mode was used to obtain the left ventricular internal diameter at end-diastole (LVIDD) and at end-systole (LVIDS), left ventricular posterior wall thickness, interventricular septal thickness (IVST), and fractional shortening (FS) in the right parasternal long-axis left ventricular outflow tract view. M-mode values were used to calculate end-diastolic volume (EDV), end-systolic volume (ESV), ejection fraction (EF), stroke volume (SV), and cardiac output (CO) by the Teicholz method. LVIDD and LVIDS were normalized for body weight to obtain normalized left ventricular internal diameter at end-diastole (LVIDDN) and normalized left ventricular internal diameter at end-systole (LVIDSN). M-mode-derived EDV and ESV were divided by body surface area (BSA) to obtain end-diastolic volume index (EDVI) and end-systolic volume index (ESVI). The BSA was calculated by the following formula (28):


$$BSA=0.101\times \text{body}\;\text{weight}\;{\left(\text{kg}\right)}^{\frac{2}{3}}$$

The stroke volume index (SVI) was calculated as SV divided by BSA. The cardiac output index (CI) was calculated as CO divided by BSA. The relative wall thickness (RWT), left ventricular mass (LVM), and left ventricular mass index (LVMI) (29) were calculated according to the following formula:


$$RWT=2\times \frac{\text{LVPWTd}}{\text{LVIDd}}$$

$$LVM=0.8\left[1.04\left\{{\left(\text{LVIDd}+\text{PWTd}+\text{IVSTd}\right)}^{3}-\text{LVID}d^{3}\right\}\right]+0.6$$

LVMI=LVM/BSA

Pulsed-wave Doppler was used to measure the peak velocity of early diastolic (E wave) and late diastolic (A wave) transmitral flow in the apical four-chamber view, wherein tissue Doppler was used to assess myocardial function by measuring the systolic (S’) and early diastolic (E’), late diastolic (A’) velocity, isovolumic relaxation time (IVRT), isovolumic contraction time (IVCT), and left ventricular ejection time (LVET) at the septal mitral annulus. The Tei index of the left ventricle was calculated as (IVRT + IVCT) / LVET and used to access left ventricular comprehensive function, including diastolic and systolic performances (30). The absolute change values were obtained by subtracting the baseline value from the value at each time point to compare absolute change in the obtained echocardiographic parameters between groups at the same time point.

### 2.5 2D-STE of LV function

The same Aplio i800 with a 6S3 sector transducer that performed conventional echocardiography was used to assess the LV myocardial function by 2D-STE. Digital clips containing at least three cardiac cycles in a B-mode were acquired and stored for further offline analysis. The LV myocardial strain was analyzed using 2D Wall Motion Tracking software (Canon Medical Systems Corporation, Otawara, Japan) embedded in the machine. Radial and circumferential strain were obtained from the right parasternal short axis at the level of the papillary muscles. Longitudinal strain was obtained from the left four-chamber apical view. The contouring of the LV myocardial border was performed at the end diastole. In the LV short axis view, the software automatically recognized the myocardium when the three points of the endocardium (4, 8, 12 o’clock) were manually inserted. For the left apical view, the software automatically recognized the LV wall following the selection of a two or four chamber view and LV was analyzed. If the borders of the LV myocardium were not accurate, the radiology resident performed a manual correction. The software’s algorithm automatically divided the LV wall into six segments and six mean strain values were obtained from each segment. These were used to calculate the global strain, including global longitudinal strain (GLS), global radial strain (GRS), global circumferential strain (GCS).

### 2.6 Statistical analysis

Statistical analysis was performed using SPSS Statistics version 20 (IBM Corp., Armonk, New York, USA). Statistical significance was defined as *p*-values <0.05 and < 0.01. The normality of the distribution was assessed using the Shapiro–Wilk test, and parametric or non-parametric techniques were applied. Parametric values are expressed as mean and standard deviation and non-parametric values are expressed as median and interquartile range (IQR).

Conventional echocardiography and 2D-STE parameters were compared between groups using the same statistical methods. Within groups, the changes in parameters from baseline were evaluated by one-way repeated-measures analysis of variance (RM ANOVA) or Friedman two-way ANOVA by ranks. The pairwise comparison at the same timepoint (baseline, T30, and T60) between two groups was performed by an independent *t*-test or Mann–Whitney *U*-test. Welch’s test was used instead of the independent *t*-test when not equally distributed. When comparing the three groups simultaneously, two-way RM ANOVA or generalized estimating equation (GEE) was performed to evaluate group differences over anesthesia time. To compare the parameters of all three groups at the same timepoint, the Kruskal–Wallis test was performed. The Mann–Whitney *U-test* was used to determine intergroup differences for two groups at the same timepoint. To clearly show the intergroup changes over time, the graph was plotted using a line plot.

Absolute changes from baseline in each group were presented as mean and 95% confidence intervals or median and IQR. Absolute changes were compared between the two groups at the same time point using an independent t-test or Mann–Whitney U-test.

## 3 Results

### 3.1 Population characteristics

The Control group comprised 8 dogs (5 males and 3 females), aged 5.5 ± 1.1 years and weighing 9.01 ± 0.76 kg. The PIMO PO group comprised 10 dogs (7 males and 3 females), aged 7.9 ± 1.86 years and weighing 8.49 ± 0.7 kg. The PIMO IV group comprised 8 dogs (4 males and 4 females), aged 9.38 ± 2 years and weighing 15.38 ± 3.46 kg.

### 3.2 Comparison of conventional echocardiographic and hemodynamic parameters

The data comparing the common parameters of the Control, PIMO PO, and PIMO IV group in the anesthetized dogs are summarized in Table 1. Additional parameters that were only compared between the Control and PIMO PO groups are described in the submitted Supplementary Table 1. Despite the different populations, the baseline values of all parameters were not statistically significantly different between groups.

**Table 1:** Hemodynamic and echocardiographic values between the Control, PIMO PO, and PIMO IV groups over anesthesia time.

	Control			PIMO PO			PIMO IV			
Parameters	Baseline^‡^	T30	T60	Baseline^‡^	T30	T60	Baseline^‡^	T30	T60	*p*-value (between all groups)
HR (bpm)	93.5 ± 17.30	88.75 ± 16.68	70.5 ± 18.46	106 ± 31.61	91.4 ± 18.76	80 ± 14.42	121 ± 28.36	99 ± 30.89	82.13 ± 20.26*	0.595
SBP (mmHg)	125 (112.75–129.25)	80 (64.5–83)	65 (60–69.5)*	125 (117.5–135)	80 (60–90)	72.5 (60–85)*	101.5 (86.25–125)	70 (70–83.75)	75 (60–90)	0.193
SVI (mL/m^2^)	46.89 ± 11.71	23.69 ± 8.86*^a^	20.91 ± 6.08*^a^	44.09 ± 11.30	25.86 ± 14.56*^a^	27.27 ± 11.41*^a^	35.22 ± 7.93	22.01 ± 6.95*	23.42 ± 10.54	0.103
CI (L/min* m^2^)	4.64 (3.14–5.65)	1.85 (1.35–2.93)	1.66 (1.07–2.02)*	4.82 (3.27–5.42)	2.30 (1.17–3.01)*^a^	1.93 (1.30–2.90)*^a†^	3.9 (3–4.9)	2.12 (1.55–2.44)	1.75 (1.38–1.88)*	0.739
ESVI (mL/m^2^)	16.27 ± 3.45	22.21 ± 7.61*^a^	33.46 ± 13.91*^b^	16.65 ± 3.28	14.65 ± 7.90	17.47 ± 11.63^†^	17.3 ± 5.85	12.46 ± 8.96^†^	9.60 ± 5.17^†^	**<0.001**
EDVI (mL/m^2^)	63.16 ± 12.74	45.90 ± 15.49*	54.37 ± 17.33	60.91 ± 12.96	39.51 ± 19.05*^a^	43.98 ± 19.63*^a^	52.52 ± 10.88	34.47 ± 14.08*^a^	33.03 ± 15.12*^a†^	0.466
LVIDSN	0.84 ± 0.07	0.94 ± 0.14*^a^	1.09 ± 0.2*^b^	0.85 ± 0.07	0.79 ± 0.16	0.83 ± 0.20^†^	0.84 ± 0.12	0.72 ± 0.19^†^	0.66 ± 0.14^†^	**<0.001**
LVIDDN	1.43 ± 0.14	1.25 ± 0.19	1.34 ± 0.18	1.41 ± 0.14	1.16 ± 0.23*^a^	1.22 ± 0.23*^a^	1.31 ± 0.12	1.10 ± 0.18*^a^	1.08 ± 0.2*^a†^	0.382
RWT	0.60 (0.53–0.73)	0.67 (0.53–0.95)	0.60 (0.50–0.69)	0.70 (0.54–0.75)	0.85 (0.63–1.22)	0.79 (0.54–1.18)	0.53 (0.5–0.73)	0.89 (0.72–1.06)	0.88 (0.77–1.15)*^†^	**0.048**
LVMI (mg/m^2^)	120.31 (114.60–132.08)	95.22 (87.66–117.33)*	105.84 (94.06–115.68)	121.2 (116.01–138.67)	106.09 (88.60–114.40)	103.03 (89.94–121.53)	122.85 (91.18–147.26)	103.66 (85.59–127.69)	100.29 (86.12–129.89)	0.908
FS (%)	41.95 (37.25–45.23)	24.5 (22.85–27.98)*^a^	17 (14.03–23.7)*^a^	38.55 (36.92–44.41)	32.26 (26.48–44.10) ^†^	34.07 (28.44–41.04) ^†^	35.35 (30.83–42.13)	35.55 (31.03–39.4)^†^	39.4 (34.33–41.03)^†^	**<0.001**
EF (%)	74.55 (69.03–78.73)	51.5 (48.35–56.83)*^a^	37.2 (31.23–50.08)*^a^	71.16 (68.61–77.15)	63.65 (53.99–77.95)^†^	65.78 (57.75–73.87)^†^	67.3 (60.93–74.63)	68 (60.38–71.8)^†^	72.35 (65.25–74.98)^†^	**<0.001**

Values are reported as mean ± standard deviation or median (interquartile range).

‡No statistically significant difference at baseline by groups.

Significant differences from baseline values are shown as *p < 0.05. Significantly different values of the PIMO PO and PIMO IV group from the Control group at the same time are shown as †*p* < 0.05. In the same row, the small superscript letters are used to compare values within the same group when both time points are significantly different from baseline. The *p* values in the table indicate statistical differences between the three groups simultaneously, with boldface indicating that the *p* value < 0.05.

T30, 30 min after induction; T60, 60 min after induction; HR, heart rate; SBP, systolic blood pressure; SVI, stroke volume index; CI, cardiac output index; ESVI, end-systolic volume index; EDVI, end-diastolic volume index; LVIDSN, normalized left ventricular internal diameter at end-systole; LVIDDN, normalized left ventricular internal diameter at end-diastole; RWT, relative wall thickness; LVMI, left ventricular mass index; FS, fractional shortening; EF, ejection fraction.

When comparing values at the same time point in the Control and PIMO PO group, FS (at T30 *p* = 0.041, at T60 *p* = 0.006) and EF (at T30 *p* = 0.033, at T60 *p* = 0.006) were significantly higher in the PO group than in the Control group. CI (*p* = 0.041) was only significantly higher in the PIMO PO group than in the Control group at T60. ESVI (*p* = 0.017), LVIDSN (*p* = 0.015), LA/Ao (*p* = 0.045), IVCT (*p* = 0.018), and LVET (*p* = 0.027) were only significantly lower in the PIMO PO group than in the Control group at T60. No significant differences at both same timepoints were found in HR, SBP, SVI, EDVI, LVIDDN, RWT, LVMI, LVIDd/Ao, E wave, A wave, E/A, E/E’, E’/A’, Tei index, S’, E’, A’, and IVRT (Table 1 and Supplementary Table 1). When comparing values at the same time point in the Control and PIMO IV group, FS (at T30 *p* = 0.009, at T60 *p* = 0.001) and EF (at T30 *p* = 0.01, at T60 *p* = 0.001) were significantly higher in the PIMO IV group than in the Control group. At both timepoints, ESVI (at T30 *p* = 0.034, at T60 *p* < 0.001) and LVIDSN (at T30 *p* = 0.019, at T60 *p* < 0.001) were significantly lower than the Control group. RWT (*p* = 0.012) were only significantly higher in the PIMO IV group compared to the Control group at T60. EDVI (*p* = 0.02) and LVIDDN (*p* = 0.015) were only significantly lower in the PIMO IV group compared to the Control group at T60. No significant differences at both same timepoints were found in HR, SBP, SVI, CI, and LVMI. When comparing values at the same time point in the PIMO PO and PIMO IV group, at both time points, no significant differences were found between the two groups on any parameter.

When the group difference between all groups over anesthesia time was evaluated, ESVI (*p* < 0.001) and LVIDSN (*p* < 0.001) showed a statistically significant increase with time in the Control group but were statistically unchanged in the PIMO PO and PIMO IV group. FS (*p* < 0.001) and EF (*p* < 0.001) showed a statistically significant decrease with time in the Control group but were statistically unchanged in the PIMO PO and PIMO IV group (Table 1).

**Figure 2:**
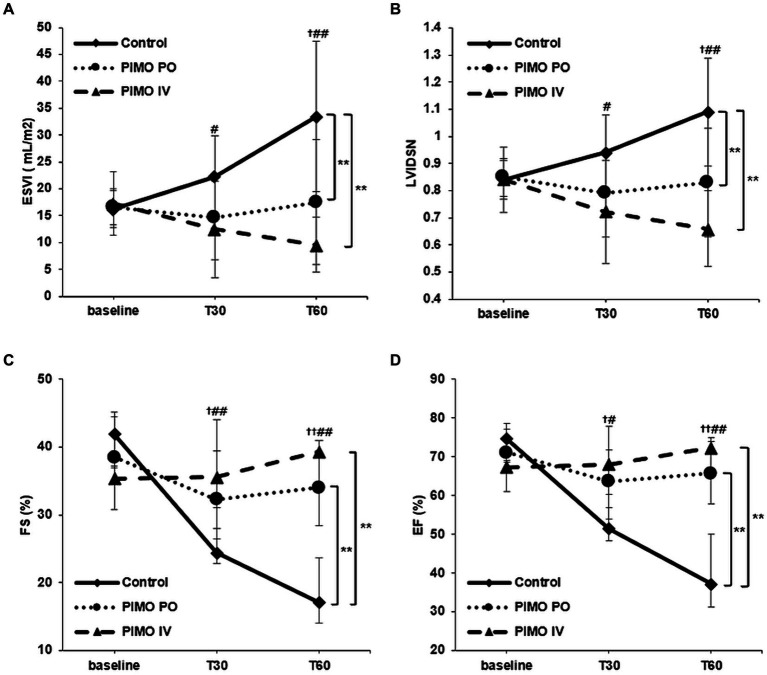
Changes in **(A)** ESVI, **(B)** LVIDSN, **(C)** FS, and **(D)** EF over time from baseline. Data are presented as mean with standard deviation **(A,B)** and medians with interquartile ranges **(C,D)**. No treatment (Control group): solid line with rhombus; Oral pimobendan group (PIMO PO group): dotted line with circle; IV pimobendan group (PIMO IV group): dashed line with triangle. ^†^*p* < 0.05 and ^††^*p* < 0.01 in comparison between the Control and PIMO PO groups. #*p* < 0.05 and ##*p* < 0.01 in comparison between Control and PIMO IV groups. ^*p* < 0.05 in comparison between the PIMO PO and PIMO IV groups. *p < 0.05 and **p < 0.01 in comparison between two groups over time by two-way RM ANOVA or GEE analysis. ESVI, end-systolic volume index; LVIDSN, normalized left ventricular internal diameter at end-systole; FS, fractional shortening; EF, ejection fraction.

Figure 2 shows the differences over anesthesia time when comparing the two groups. In ESVI (Figure 2A), the values increased significantly over anesthesia time in the Control group. However, in the PIMO PO (*p* = 0.008) and PIMO IV (*p* < 0.001) groups, the trend was significantly different from the Control group because there is no significant increase of ESVI during anesthesia time. In LVIDSN (Figure 2B), the values increased significantly over anesthesia time in the Control group. However, in the PIMO PO (*p* = 0.004) and PIMO IV (*p* < 0.001) groups, the trend was significantly different from the Control group because there is no significant increase of LVIDSN during anesthesia time. In FS (Figure 2C), the values decreased significantly over anesthesia time in the Control group. However, in the PIMO PO (*p* < 0.001) and PIMO IV (*p* < 0.001) groups, the trend was significantly different from the Control group because there is no significant decrease of FS during anesthesia time. In EF (Figure 2D), the values decreased significantly over anesthesia time in the Control group. However, in the PIMO PO (*p* < 0.001) and PIMO IV (*p* < 0.001) groups, the trend was significantly different from the Control group because there is no significant decrease of EF during anesthesia time. No significant differences were observed between the PIMO PO and PIMO IV groups in any parameters (ESVI *p* = 0.158, LVIDSN *p* = 0.162, FS *p* = 0.373, EF *p* = 0.271).

### 3.3 Comparison of absolute changes from the baseline

In the PIMO PO group, ESVI (both T30 and T60) and LVIDSN (both T30 and T60) showed a significantly smaller increase with anesthesia time than the Control group, while FS (T60) and EF (T60) showed a significantly smaller decrease with anesthesia time. In the PIMO IV group, SVI (T60), RWT (T60), FS (T30), and EF (T30) showed a significantly smaller decrease with anesthesia time than Control group. There were no significant differences in absolute changes in any parameter between the PIMO PO and PIMO IV groups (Table 2).

**Table 2:** Absolute changes from the baseline at each measurement time in the Control, PIMO PO, and PIMO IV groups.

Parameters	Time	Control	PIMO PO	PIMO IV	*p*-value (Control vs. PO)	*p*-value (Control vs. IV)	*p*-value (PO vs. IV)
HR (bpm)	T30	−4.75 (−22.36 to +12.86)	−14.6 (−38.63 to +9.43)	−22 (−46.61 to +2.61)	0.481	0.199	0.631
	T60	−28.5 (−38.5 to −15.25)	−18 (−28.25 to −12)	−25.5 (−67.5 to −25)	0.350	0.562	0.098
SBP (mmHg)	T30	−42.5 (−47 to −31.75)	−36 (−75 to −27.75)	−26.5 (−47.5 to −15)	0.754	0.188	0.197
	T60	−57.5 (−76.79 to −38.21)	−51.8 (−70.9 to −32.7)	−28.5 (−55.08 to −1.92)	0.640	0.056	0.110
SVI (mL/m^2^)	T30	−23.19 (−33.42 to −12.97)	−19.67 (−29.05 to −10.29)	−13.21 (−18.37 to −8.06)	0.568	0.058	0.191
	T60	−29.06 (−29.61 to −25.42)	−17.38 (−28.83 to −10.21)	−15.36 (−18.12 to −11.15)	0.131	**0.009**	0.286
CI (L/min* m^2^)	T30	−2.19 (−3.53 to −0.68)	−2.89 (−3.83 to −1.42)	−2 (−2.53 to −0.91)	0.374	0.600	0.248
	T60	−2.83 (−3.93 to −1.72)	−2.42 (−3.56 to −1.28)	−2.01 (−3.2 to −0.81)	0.574	0.253	0.572
ESVI	T30	+5.94 (+1.59 to +10.28)	−2 (−7.56 to +3.56)	−4.84 (−14.62 to +4.94)	**0.025**	**0.039**	0.545
	T60	+17.19 (+7.73 to +26.66)	+0.82 (−7.67 to +9.32)	−7.7 (−15.06 to −0.34)	**0.009**	**<0.001**	0.111
EDVI	T30	−17.26 (−30 to −4.52)	−21.67 (−31.8 to −11.54)	−18.05 (−31.58 to −4.53)	0.534	0.921	0.620
	T60	−6.76 (−22.04 to +2.24)	−20.22 (−29.51 to −7.72)	−24.32 (−29.22 to −16.91)	0.131	0.093	0.722
LVIDSN	T30	+0.1 (+0.02 to +0.18)	−0.06 (−0.18 to +0.06)	−0.12 (−0.32 to +0.07)	**0.025**	**0.033**	0.509
	T60	+0.26 (+0.13 to +0.38)	−0.02 (−0.17 to +0.13)	−0.18 (−0.35 to −0.02)	**0.008**	**<0.001**	0.110
LVIDDN	T30	−0.18 (−0.33 to −0.04)	−0.24 (−0.37 to −0.12)	−0.22 (−0.37 to −0.06)	0.463	0.705	0.749
	T60	−0.06 (−0.23 to +0.02)	−0.19 (−0.34 to −0.07)	−0.32 (−0.34 to −0.19)	0.131	0.036	0.450
RWT	T30	+0.09 (−0.1 to +0.28)	+0.16 (−0.02 to +0.34)	+0.28 (+0.07 to +0.49)	0.520	0.127	0.334
	T60	−0.01 (−0.16 to +0.13)	+0.1 (−0.08 to +0.28)	+0.33 (+0.08 to +0.57)	0.303	**0.015**	0.102
LVMI	T30	−20.57 (−36.46 to −4.68)	−19.54 (−39.62 to +0.53)	−13.18 (−28.08 to +1.73)	0.931	0.436	0.585
	T60	−18.71 (−29.85 to −5.41)	−33.22 (−36.49 to +9.67)	−6.81 (−32.32 to −2.13)	0.477	0.401	0.790
FS (%)	T30	−16.18 (−20.35 to −12)	−7.42 (−16.44 to +1.6)	−0.79 (−9.85 to +8.27)	0.084	**0.005**	0.256
	T60	−22.3 (−26.37 to −18.23)	−7.27 (−15.75 to +1.21)	+2.89 (−6.81 to +12.59)	**0.003**	**0.000**	0.087
EF (%)	T30	−21.8 (−26.9 to −16.7)	−9.72 (−21.09 to +1.65)	−0.78 (−13.07 to +11.52)	0.060	**0.004**	0.238
	T60	−38.3 (−40.43 to −24.48)	−3.35 (−22.98 to +2.18)	+5.05 (−8.88 to +14.05)	**0.013**	**0.001**	0.091

Absolute changes from baseline are reported as mean (95% confidence interval) or median (interquartile range).

The *p* values in the table indicate statistical differences between the two groups (Control vs PO; Control vs IV; PO vs IV), with boldface indicating that the *p* value < 0.05.

T30, 30 min after induction; T60, 60 min after induction; HR, heart rate; SBP, systolic blood pressure; SVI, stroke volume index; CI, cardiac output index; ESVI, end-systolic volume index; EDVI, end-diastolic volume index; LVIDSN, normalized left ventricular internal diameter at end-systole; LVIDDN, normalized left ventricular internal diameter at end-diastole; RWT, relative wall thickness; LVMI, left ventricular mass index; FS, fractional shortening; EF, ejection fraction.

### 3.4 Comparison of LV strain values using 2D-STE

Changes in the values of the speckle tracking indices, such as GLS, GRS, and GCS, by anesthesia time and group are summarized in Table 3. When the group difference between all groups over anesthesia time was evaluated, GRS only showed a statistically significant decrease with time in the Control group but was statistically unchanged in the PIMO PO (*p* = 0.009) and PIMO IV group (*p* = 0.024). There was no significant difference between the PIMO PO and PIMO IV groups (*p* = 0.177). GLS showed no significant decrease in the PIMO PO (*p* = 0.034) and PIMO IV groups (*p* = 0.042) as compared to the Control group, with a tendency for baseline values to be maintained. However, the group differences were not statistically significant (*p* = 0.55). GCS showed declining trends in the Control and PIMO PO groups, but not in the PIMO IV group. However, no significant differences were obtained (*p* = 0.599). At the same time points, GLS was significantly higher at both time points (at T30 *p* = 0.016, at T60 *p* = 0.025) compared to the Control group, and GRS was higher at T60 (*p* = 0.009) in the PIMO PO group. In the PIMO IV group, GLS was significantly higher at both time points (at T30 *p* = 0.034, at T60 *p* = 0.023), and GRS was higher at T60 (*p* = 0.041) compared to the Control group. There was no significant difference between the PIMO PO and PIMO IV groups at the same time points.

**Table 3:** Global LV strain values measured by 2D speckle-tracking echocardiography for the Control, PIMO PO, and PIMO IV groups.

	Control			PIMO PO			PIMO IV			
Strain value	Baseline^‡^	T30	T60	Baseline^‡^	T30	T60	Baseline^‡^	T30	T60	*p*-value (between all groups)
GLS (%)	−14.85 ± 2.99	−9.31 ± 2.42*^a^	−8.62 ± 2.19*^a^	−17.12 ± 4.49	−13.56 ± 2.21^†^	−14.23 ± 3.56^†^	−18.59 ± 4.78	−14.24 ± 3.06^†^	−14.77 ± 3.74^†^	0.55
GRS (%)	41.88 ± 3.44	29.26 ± 3.04*^a^	27.77 ± 2.51*^a^	41.52 ± 2.77	33.74 ± 4.22	34.79 ± 5.85^†^	46.81 ± 7.88	36.89 ± 8.29	37.28 ± 7.16^†^	**0.015**
GCS (%)	−18.56 ± 3.43	−13.64 ± 3.18	−12.85 ± 1.77	−19.16 ± 2.89	−13.53 ± 2.83*^a^	−13.72 ± 4.16*^a^	−18.17 ± 4.51	−15.58 ± 2.18	−16.27 ± 3.48	0.599

Strain values are reported as mean with standard deviation.

‡No statistically significant difference at baseline by groups.

Significant differences from baseline values are shown as *p < 0.05. Significantly different values of the PIMO PO and PIMO IV group from the Control group at the same time are shown as †*p* < 0.05. In the same row, the small superscript letters are used to compare values within the same group when both time points are significantly different from baseline. The *p* values in the table indicate statistical differences between the three groups simultaneously, with boldface indicating that the *p* value < 0.05.

### 3.5 Adverse events

No adverse event was identified in any of the dogs in the study, in both short-term (24 h) and long-term (3 days) monitoring. There were no significant clinical symptoms, such as vomiting.

## 4 Discussion

This study demonstrated the changes in hemodynamic and echocardiographic parameters following pimobendan administration orally or intravenously to dogs prior to anesthesia, and compared them to dogs no receiving pimobendan. Of all parameters, FS, EF, and GRS significantly decreased, and ESVI and LVIDSN significantly increased with anesthesia time in the Control group compared to the PIMO (PO and IV) group. In the both PIMO groups, no significant changes were observed over time. These differences suggest that single-dose oral pimobendan administration before anesthesia could be beneficial to preserve systolic function and induce effects similar to pimobendan IV injection.

In this study, a significant time-dependent increase in ESVI, LVIDSN, IVCT, and IVRT, and a decrease in SVI, FS, EF, E wave, and A wave from baseline were observed in the Control group. These changes reflect the decreased systolic function, decreased myocardial contractility, and impaired relaxation of the LV during anesthesia by propofol and isoflurane, and match the findings of previous studies (27, 31, 32). Of these parameters, ESVI, LVIDSN, FS, EF, and E wave remained statistically unchanged from the baseline in the PIMO PO group compared to the Control group. Pimobendan has a dual mechanism of action, causing myocardial contraction by calcium sensitization, a positive inotropic effect, and vasodilator effects by inhibiting phosphodiesterase 3 (6). The positive inotropic effect of pimobendan is associated with reduced ESVI, LVIDSN and increased FS and EF. Furthermore, by reducing LV end-diastolic pressure (33), pimobendan may have caused an active relaxation of the LV myocardium, resulting in an increased E wave. These results show that a single-dose of oral pimobendan prior to anesthesia has a sufficient effect on cardiac systolic function and LV relaxation.

Compared to the values of the Control group at the same timepoint, the PIMO PO group showed significant differences in FS and EF at T30, and in CI, ESVI, LVIDSN, FS, EF, LA/Ao, IVCT, and LVET at T60. This suggests that the effect of oral pimobendan was enhanced up to 90 min after administration, which is consistent with previous studies on the pharmacokinetics of oral pimobendan (34, 35). In one study, maximal cardiovascular effects were observed 2–4 h after a single oral dose (0.26–0.28 mg/kg) in healthy dogs (35) whereas, in another study, the mean time to peak concentration with a single oral dose of pimobendan in dogs with MMVD was 1.66 h, independent of the ACVIM stage (34).

The effect of pimobendan on systolic function was again confirmed in the PIMO IV group, wherein the parameters associated with systolic function, such as ESVI, LVIDSN, FS, and EF, were maintained during the anesthesia time without significant changes from baseline. In the PIMO IV group, significant reductions from baseline in SVI, CI, EDVI, and LVIDDN were observed. In a previous study, when Lactated Ringer’s solution was infused at a rate of 5 mL/kg/h during anesthesia, SVI and CI increased at 15 min after a single dose of pimobendan IV (0.15 mg/kg) (11). In our study, despite the injection of intravenous pimobendan, no significant differences in SVI or CI from the Control group were observed, possibly be due to the absence of fluid therapy. EDVI and LVIDDN were significantly lower in the PIMO IV group than in the Control group, which is consistent with the results of previous studies that pimobendan reduces LV diastolic diameter (14, 16, 35, 36). The finding that significant differences in EDVI and LVIDDN were observed only in the PIMO IV group and not the PIMO PO group, may indicate that intravenous administration is more effective. In a previous study comparing the pharmacokinetics of oral and intravenous pimobendan, intravenous pimobendan showed a greater maximal concentrations of pimobendan (37).

When the interactions of anesthesia time and group were evaluated across the three groups simultaneously, significant group differences were found in ESVI, LVIDSN, FS, and EF. ESVI, LVIDSN, FS, and EF are used to evaluate cardiac systolic function. FS and EF are affected by various factors such as preload, afterload, contractility, and heart rate, while ESVI and LVIDSN are mainly affected by contractility and afterload (38). End systolic parameters such as ESVI and LVIDSN have predictive value for congestive heart failure and survival (39–42). Compared to the Control group, ESVI and LVIDSN remained lower without significant change from baseline, and EF and FS remained higher without significant change from baseline in the PIMO PO and PIMO IV groups over anesthesia time. These findings suggest that pimobendan primarily affects LV systolic function and contractility, which is consistent with the main effects of pimobendan identified in previous studies (6, 11, 13, 15, 16, 33, 35, 43, 44). A significant maintenance of LV contractility was observed with both oral and intravenous pimobendan.

For absolute changes, significant differences from the Control group were observed in SVI and RWT in the PIMO IV group compared to the PIMO PO group, and significant differences were observed in T30 for the same indicators, such as FS and EF. These results also suggest that the effectiveness of the intravenous route may be higher for some parameters than the oral route (37), consistent with the results of absolute changes. However, no significant differences were found between the PIMO PO and PIMO IV groups in ESVI, LVIDSN, FS and EF at the same timepoints, and group differences by anesthesia time were not statistically significant. Absolute changes at the same time point were not significantly different between the two groups. These results suggest that oral pimobendan administration 30 min before the induction of anesthesia showed a similar preservation of LV systolic function during 60 min of anesthesia as intravenous pimobendan injection, 10 min after induction.

Assessment of LV systolic function using FS and EF derived from conventional echocardiography is highly hemodynamic load-dependent (45). FS does not reflect all longitudinal, radial, and circumferential myocardial movements of the LV myocardium. The global strain is calculated as the average of the segmental strains or the average of the strains of the many speckles in the myocardium area. It can represent the entire myocardial region (18). In the present study, significant reduction of GLS and GRS was identified in the Control group. A declining trend was observed in GCS, although statistical significance was not obtained. These results may suggest that myocardial functions in all directions were reduced under general anesthesia. Longitudinal strain is mainly affected by the subendocardial region and is vulnerable to hypoperfusion or ischemic injury (46). Radial strain was a useful indicator of contractility along with circumferential strain and mainly governed by midmyocardial region (47). Global strain analysis showed that oral and intravenous pimobendan administration was able to sustain myocardial motion in both the longitudinal and radial directions. This is consistent with previous findings on improvements in left atrial and right ventricular strain with pimobendan treatment (11, 43, 48). Therefore, these results suggest that even a single administration of oral and intravenous pimobendan could improve myocardial contractility and perfusion in anesthetized dogs.

Finally, no significant adverse effects, such as vomiting from pre-anesthetic administration, were identified in this study. These results suggest that a single dose of oral pimobendan before anesthesia was safe. In a previous study comparing adverse events between placebo and pimobendan groups in 359 dogs with MMVD, no significant differences in adverse events, such as diarrhea, vomiting, and anorexia were found between the two groups (49). The target dose of pimobendan in this previous study was 0.2–0.3 mg/kg every 12 h, which is similar to the single-dose in our study.

This study had a few limitations. First, the small sample size and different populations might have decreased the statistical significance of the differences over time and groups. Additionally, the individual influence of subclinical regurgitation and the different period between anesthesia were not considered. Second, some previous studies have used invasive techniques, such as micromanometer-tip catheters to assess LV pressure or thermodilution techniques to assess CO (33, 49), which were not appropriate for this study because they involved owner-owned dogs. Third, this study only performed anesthesia for 1 h; the results of this study are not entirely applicable to longer anesthesia. Therefore, we expect that further studies with larger populations and/or longer durations of anesthesia may produce beneficial results. Lastly, E wave showed significantly different interactions between the Control and PIMO PO groups, but statistical analysis of the three groups could not be performed because some dogs in the PIMO IV group had only the parameters of M-mode echocardiography.

In conclusion, pre-anesthetic administration of oral pimobendan is beneficial for maintaining cardiac systolic function and LV myocardial contractility during general anesthesia, as determined by measuring changes in various hemodynamic and echocardiographic parameters. Oral pimobendan has the advantage of being more readily available, less expensive, and less invasive than intravenous pimobendan. This study suggests that pre-anesthetic oral pimobendan may be a new option for dogs who require diagnostic tests and surgery under general anesthesia to preserve LV systolic function, as well as for dogs who are concerned about complications from circulatory failure.

## Data availability statement

The raw data supporting the conclusions of this article will be made available by the authors, without undue reservation.

## Ethics statement

The animal studies were approved by Institutional Animal Care and Use Committee of Konkuk University (KUIACUC-KU18073, 2018) and Jeonbuk National University (JBNUNON2022-083, 2022). The studies were conducted in accordance with the local legislation and institutional requirements. Written informed consent was obtained from the owners for the participation of their animals in this study.

## Author contributions

YJ: Writing – review & editing, Writing – original draft, Methodology, Formal analysis, Data curation, Conceptualization. BK: Writing – review & editing, Writing – original draft, Methodology, Formal analysis, Data curation, Conceptualization. S-SK: Writing – review & editing, Investigation, Formal analysis. KL: Writing – review & editing, Validation, Supervision, Project administration, Methodology, Formal analysis, Data curation, Conceptualization. HY: Writing – review & editing, Writing – original draft, Validation, Supervision, Project administration, Methodology, Investigation, Formal analysis, Data curation, Conceptualization.

## Glossary

2D-STE: two-dimensional speckle-tracking echocardiography
ANOVA: analysis of variance
A wave: peak velocity of late diastolic transmitral flow
A’: late diastolic velocity of septal mitral annulus
BSA: body surface area
CHF: congestive heart failure
CI: cardiac output index
CO: cardiac output
DCM: dilated cardiomyopathy
E wave: peak velocity of early diastolic transmitral flow
E/A: the ratio of peak velocity of early diastolic transmitral flow to peak velocity of late diastolic transmitral flow
E/E’: the ratio of peak velocity of early diastolic transmitral flow to early diastolic velocity of septal mitral annulus
E’: early diastolic velocity of septal mitral annulus
E’/A’: the ratio of early diastolic velocity to late diastolic velocity of septal mitral annulus
EDV: end-diastolic volume
EDVI: end-diastolic volume index
EF: ejection fraction
ESV: end-systolic volume
ESVI: end-systolic volume index
EtCO_2_: expiratory terminal partial pressure of carbon dioxide
FS: fractional shortening
GCS: global circumferential strain
GEE: generalized estimating equation
GRS: global radial strain
GLS: global longitudinal strain
HR: heart rate
IQR: interquartile range
IVCT: isovolumic contraction time
IVRT: isovolumic relaxation time
IVST: interventricular septal thickness
LA/Ao: left atrial-to-aortic root ratio
LV: left ventricle
LVET: left ventricular ejection time
LVOT: left ventricular outflow tract
LVFWT: left ventricular free wall thickness
LVIDD: left ventricular internal diameter at end-diastole
LVIDd/Ao: left ventricular internal diameter at end-diastole-to-aortic root ratio
LVIDDN: normalized left ventricular internal diameter at end-diastole
LVIDS: left ventricular internal diameter at end-systole
LVIDSN: normalized left ventricular internal diameter at end-systole
LVM: left ventricular mass
LVMI: left ventricular mass index
MAC: minimum alveolar concentration
MMVD: myxomatous mitral valve disease
PIMO IV: intravenous administration of pimobendan
PIMO PO: oral administration of pimobendane
PW: pulsed wave
PWT: posterior wall thickness
RM ANOVA: repeated-measures analysis of variance
RWT: relative wall thickness
S’: systolic velocity of septal mitral annulus
SBP: systolic blood pressure
SpO_2_: percutaneous oxygen saturation
SV: stroke volume
SVI: stroke volume index
T30: 30 min after induction
T60: 60 min after induction
