# Supplementary material for: Changes in echocardiographic indices and left ventricular strain values by two-dimensional speckle-tracking echocardiography following pre-anesthetic oral pimobendan administration compared with intravenous pimobendan in dogs
Source: Front Vet Sci. 2024 Jun 3;11:1394896. doi: 10.3389/fvets.2024.1394896 (PMC11184225; doi:10.3389/fvets.2024.1394896)
Supplement: Supplementary file 1 [file Table_1.DOCX]

**Supplementary Table 1. Additional echocardiographic parameters between the Control and PIMO PO groups**

|  | **Control** | | | | **PIMO PO** | | |  |
| --- | --- | --- | --- | --- | --- | --- | --- | --- |
| **Parameters** | **Baseline‡** | **T30** | **T60** | **Baseline‡** | | **T30** | **T60** | ***P*-value (Control vs PIMO PO)** |
| LA/Ao | 1.14  (1.01–1.22) | 1.11  (1.00–1.19) | 1.07  (1.07–1.17) | 1.15 (0.96–1.25) | | 1.03  (0.84–1.16)*^a^ | 1.05  (0.90–1.09)*^a†^ | 0.183 |
| LVIDd/Ao | 1.92 ± 0.18 | 1.78 ± 0.25 | 1.86 ± 0.18 | 1.79 ± 0.29 | | 1.60 ± 0.34*^a^ | 1.64 ± 0.40*^a^ | 0.372 |
| E wave (cm/s) | 76.15  (64.05–86.48) | 60.35  (44–63.48)*^a^ | 47.8  (42–63.73)*^a^ | 72.95 (62.7–85.83) | | 69.3 (44.35–84.25) | 63.15 (49.88–74.43)* | **0.011** |
| A wave (cm/s) | 54.55  (40.18–70.6) | 25.4  (20.04–32.9)*^a^ | 25.85  (18.01–33.85)*^a^ | 58.7  (41.13–69.75) | | 24  (17.33–30.7)*^a^ | 25.2  (18.68–31.73)*^a^ | 0.663 |
| E/A | 1.29 (0.92–1.95) | 2.32 (1.61–3.05)* | 2.17 (1.32–2.79) | 1.17 (0.96–1.68) | | 2.42  (2.01–4.01)*^a^ | 2.38  (1.63–3.19)*^a^ | 0.099 |
| E/E' | 10.71 ± 2.36 | 12.52 ± 2.63 | 11.57 ± 3.09 | 10.54 ± 2.12 | | 11.98 ± 3.37 | 13.51 ± 5.40 | 0.304 |
| E'/A' | 1.18 ± 0.40 | 1.53 ± 0.42* | 1.46 ± 0.54 | 1.10 ± 0.39 | | 1.59 ± 0.66 | 1.41 ± 0.64 | 0.778 |
| Tei index | 0.68 (0.53–0.8) | 0.71 (0.59–1.19) | 0.83 (0.69–0.96) | 0.68 (0.53–0.78) | | 0.66 (0.61–0.99) | 0.68 (0.56–0.78) | 0.445 |
| S' (cm/s) | 7.77 ± 1.66 | 4.3 ± 0.54*^a^ | 4.34 ± 1.14*^a^ | 8.24 ± 2.23 | | 6.08 ± 3.02 | 6.15 ± 2.38 | 0.369 |
| E' (cm/s) | 7.04 ± 1.73 | 4.71 ± 0.86*^a^ | 4.54 ± 1.14*^a^ | 7.03 ± 1.62 | | 5.76 ± 2.62 | 5.05 ± 2.31* | 0.263 |
| A' (cm/s) | 6.24 (5.33–7.56) | 3.34  (2.94–3.49)*^a^ | 3.45  (2.88–3.78)*^a^ | 6.45 (5.58–7.96) | | 3.55  (2.95–4.44)*^a^ | 3.41  (3.1–3.99)*^a^ | 0.971 |
| IVCT (ms) | 46.80  (37.75–48.00) | 55  (50–78.75) | 67.5  (55.25–84)* | 44.5 (35.25–48) | | 52 (33.25–65.75) | 48.5 (31–60) ^†^ | 0.059 |
| IVRT (ms) | 53  (46.85–80) | 70  (50–105.25) | 90  (71.75–98.25)* | 53 (45.75–75.5) | | 62.5 (54.25–87.5) | 70 (48.75–80) | 0.479 |
| LVET (ms) | 164.25 ± 19.36 | 167 ± 22.11 | 189.63 ± 17.25 | 158 ± 23.35 | | 151.8 ± 25.32 | 168.7 ± 18.80^†^ | 0.57 |

Values are reported as mean ± standard deviation or median (interquartile range).

‡No statistically significant difference at baseline by groups.

Significant differences from baseline values are shown as *P < .05. Significantly different values of the PIMO PO group from the Control group at the same time are shown as †P < .05. In the same row, the small superscript letters and the large superscript letters are used to compare values within the same group when both time points are significantly different from baseline. The bold P-values for interaction groups and time indicate <.05.

T30, 30 minutes after induction; T60, 60 minutes after induction; LA/Ao, left atrial-to-aortic root ratio; LVIDd/Ao, left ventricular internal diameter at end-diastole-to-aortic root ratio; E wave, peak velocity of early diastolic transmitral flow; A wave, peak velocity of late diastolic transmitral flow; E/A, the ratio of peak velocity of early diastolic transmitral flow to peak velocity of late diastolic transmitral flow; E/E’, the ratio of peak velocity of early diastolic transmitral flow to early diastolic velocity of septal mitral annulus; E’/A’, the ratio of early diastolic velocity to late diastolic velocity of septal mitral annulus; S’, systolic velocity of septal mitral annulus; E’, early diastolic velocity of septal mitral annulus; A’, late diastolic velocity of septal mitral annulus; IVCT, isovolumic contraction time; IVRT, isovolumic relaxation time; LVET, left ventricular ejection time.
